# A novel method for dynamically altering the surface area of intracranial EEG electrodes

**DOI:** 10.1088/1741-2552/acb79f

**Published:** 2023-03-07

**Authors:** Kavyakantha Remakanthakurup Sindhu, Duy Ngo, Hernando Ombao, Joffre E Olaya, Daniel W Shrey, Beth A Lopour

**Affiliations:** 1 Department of Biomedical Engineering, University of California, Irvine, Irvine, CA, United States of America; 2 Department of Statistics, Western Michigan University, Kalamazoo, MI, United States of America; 3 Statistics Program, King Abdullah University of Science and Technology, Thuwal, Saudi Arabia; 4 Division of Neurosurgery, Children’s Hospital of Orange County, Orange, CA, United States of America; 5 Department of Neurosurgery, University of California, Irvine, Irvine, CA, United States of America; 6 Division of Neurology, Children’s Hospital of Orange County, Orange, CA, United States of America; 7 Department of Pediatrics, University of California, Irvine, Irvine, CA, United States of America

**Keywords:** electrocorticogram, electrode properties, electrode model, electrode size, interictal epileptiform discharge, power spectrum, intracranial electroencephalogram

## Abstract

*Objective.* Intracranial electroencephalogram (iEEG) plays a critical role in the treatment of neurological diseases, such as epilepsy and Parkinson’s disease, as well as the development of neural prostheses and brain computer interfaces. While electrode geometries vary widely across these applications, the impact of electrode size on iEEG features and morphology is not well understood. Some insight has been gained from computer simulations, as well as experiments in which signals are recorded using electrodes of different sizes concurrently in different brain regions. Here, we introduce a novel method to record from electrodes of different sizes in the exact same location by changing the size of iEEG electrodes after implantation in the brain. *Approach.* We first present a theoretical model and an *in vitro* validation of the method. We then report the results of an *in vivo* implementation in three human subjects with refractory epilepsy. We recorded iEEG data from three different electrode sizes and compared the amplitudes, power spectra, inter-channel correlations, and signal-to-noise ratio (SNR) of interictal epileptiform discharges, i.e. epileptic spikes. *Main Results.* We found that iEEG amplitude and power decreased as electrode size increased, while inter-channel correlation did not change significantly with electrode size. The SNR of epileptic spikes was generally highest in the smallest electrodes, but 39% of spikes had maximal SNR in larger electrodes. This likely depends on the precise location and spatial spread of each spike. *Significance.* Overall, this new method enables multi-scale measurements of electrical activity in the human brain that can facilitate our understanding of neurophysiology, treatment of neurological disease, and development of novel technologies.

## 1. Introduction

Intracranial electroencephalogram (iEEG) is an invasive technique that measures electrical activity of the brain. It is used for the diagnosis, monitoring, and treatment of neurological diseases, such as epilepsy [1] and Parkinson’s disease [2]. It has also been critical to the development of devices such as neural prostheses [3] and brain computer interfaces (BCIs) [4]. iEEG measurement is done using subdural grids or strips of electrodes placed on the surface of the cerebral cortex or depth electrodes inserted into brain tissue. The signals measured by iEEG electrodes reflect the aggregate electrical activity of the cortical neurons in the immediate vicinity [5]. The voltage measured by an electrode is thought to reflect the average potential distribution under its uninsulated contact area [6–8]. The signals recorded by a subdural grid depend on a number of factors, including the impedance, geometry, and spacing of the electrodes [5].

Because the number of neurons whose electrical activity contribute to the iEEG signal is proportional to the electrode contact area, electrode size is an important factor in measurements. A multitude of electrodes of different geometries are used for intracranial EEG measurement. Penetrating microwires with diameters as low as 12 *μ*m have been used for *in vivo* single unit recordings [9, 10]. Micro-electrocorticography electrodes, which have potential uses in both BCI and clinical applications, have diameters in the range of 10 *μ*m to several hundreds of micrometers [10, 11]. Standard clinical macroelectrodes for iEEG have exposed diameters that range from 0.86 mm to 3 mm [12]. Therefore, it is critical to understand the precise relationship between electrode size and iEEG measurement to better interpret and compare the results of studies with different methodologies.

*In silico* studies of the effect of electrode size on iEEG signal characteristics present conflicting pictures. Nelson and Pouget [13] developed a physical model that predicted electrodes with larger surface area would have higher correlation between them. Their model also suggested that, as the voltage profile underneath the electrode becomes more inhomogeneous (as it would with increasing surface area), electrodes with different contact areas are more likely to measure different average values. Ollikainen *et al* [6] simulated rectangular electrodes with surface areas that varied from 1.5 cm^2^ to 5 cm^2^, measuring electrical potential from a single source. They showed that smaller electrodes had more sensitivity to localized voltage differences than larger electrodes. This is consistent with the idea that each electrode measures the average potential of the underlying tissue; therefore, using larger electrodes results in loss of spatial information. Furthermore, these simulations showed that the current distribution on the surface of the electrode is non-uniform and concentrated at the boundaries, with the distribution becoming more complex when larger electrodes are used. Moffitt and McIntyre [14] developed a model demonstrating that smaller contacts exhibited higher signal amplitude when neurons were close to the electrode. Contrary to this, the model developed by Suihko *et al* [15] suggested that changing the electrode size will cause only small changes in the sensitivity distribution and is therefore not a key factor in iEEG measurements. The model of Lempka *et al* [16] also suggested that the size of the recording micro-electrode does not have much effect on signal amplitude, while impedance does.

A number of studies using penetrating microelectrodes have analyzed neuronal action potentials and the effect of electrode size on their measurement [17–20]. Anderson *et al* posited that, in the context of action potentials, as the size of the electrode increases, the ‘listening sphere’ increases, but the signal-to-noise ratio (SNR) decreases [18]. However, a study by Ward *et al* in an animal model found no significant difference in the action potential SNRs for implanted micro-electrode arrays of different surface areas [19]. The results of such studies can be confounded by differences in the electrode coating and other techniques to lower the electrode impedance, independent of the electrode diameter.

In contrast, there are few *in vivo* studies analyzing the effect of electrode size on general iEEG characteristics, such as amplitude or waveform morphology [21, 22]. In applications like BCI and neuro-prosthetic devices, the ability to accurately classify neural signals associated with different cognitive tasks is of utmost importance. Studies in this field have shown that smaller electrode size and higher grid density (e.g. 100 *μ*m diameter and 1 mm pitch) enable the recording of signals from smaller spatial scales, making them more suitable for these applications [22–24]. In human studies of epilepsy, various electrode sizes have been used to measure high frequency oscillations (HFOs), a candidate biomarker for epileptogenic brain tissue. HFOs are highly localized, transient iEEG events characterized by high-amplitude 80–500 Hz oscillations. Using human intracranial EEG, Chatillon *et al* [25] analyzed HFOs recorded with electrodes of different sizes (ranging from 0.02 to 0.09 mm^2^) and found that the difference in recordings was not clinically relevant. On the other hand, Worrell *et al* reported that smaller electrodes recorded more HFOs than larger electrodes (diameter of 40 *μ*m, as opposed to 2.3 mm), particularly in the 250–500 Hz frequency range [26]. Another study by Boran *et al* found that intraoperative HFO measurement is aided by the use of an electrode grid with smaller electrodes and higher density (exposed diameter of 2.3 mm and inter-electrode distance of 5 mm), compared to a standard ECoG grid with 5 mm diameter and 10 mm spacing [27]. It has also been shown that discharges resembling interictal epileptiform activity, but confined to much smaller spatial scales, can be seen using micro-electrodes but not standard-size electrodes [28, 29]. Knowledge of the relationship between electrode size and iEEG biomarker features would inform epilepsy surgery and invasive monitoring, with the potential to improve patient outcomes.

Therefore, the goal of this study was to directly measure the impact of electrode surface area on iEEG signal characteristics in the human brain. Previous work in humans has relied on simultaneous iEEG recordings using electrodes of different sizes, with each electrode implanted in a different location. In those cases, it is not clear if the resulting differences are due to electrode size or regional differences in brain activity. An alternative methodology is to record sequential iEEG measurements using electrodes of multiple sizes, placed over the exact same region of neural tissue. However, because implantation of intracranial electrodes is an invasive procedure with inherent risk to the patient, this presents logistical and ethical challenges. Here, we present a solution to this problem: a method to alter the size of an intracranial recording electrode after implantation. This enables multiscale measurements from a single region of the brain, allowing for a more direct comparison of iEEG signals recorded using electrodes of different sizes. We also examine how electrode size affects basic iEEG properties, such as power spectrum and amplitude, and we explore the effect on the morphology of interictal epileptiform discharges (also called epileptic ‘spikes’), which is a common electrographic event in epilepsy patients.

## 2. Theoretical basis for altering electrode size via electrical shorting

Our method to alter the surface area of an implanted grid electrode involves electrically connecting adjacent electrodes together (via physical shorting) to generate a range of effective surface areas. For each individual electrode, the recorded signal reflects the average voltage across the uninsulated surface; therefore, connecting two adjacent electrodes will report the average of the two individual electrodes, equivalent to doubling the electrode surface area [7, 8]. In this way, we can alter electrode size while recording data from the exact same region of neural tissue within the framework of standard clinical care.

**Figure 1.**
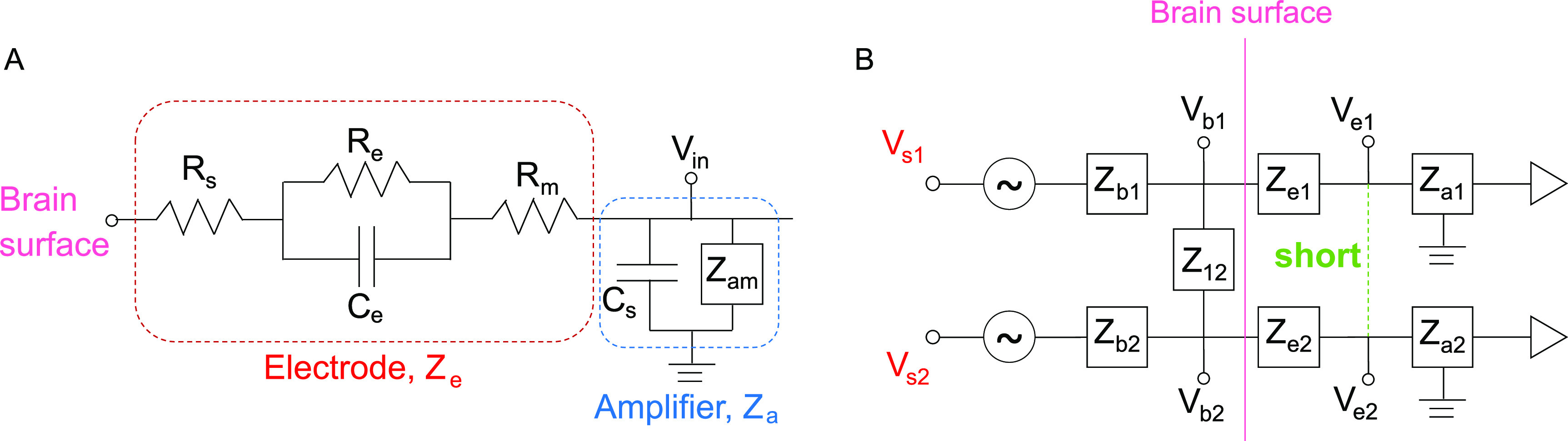
(A) Circuit model of a single electrode (red box) connected to an ideal amplifier (blue box). (B) Circuit model of two electrodes (*Z*_e1_, *Z*_e2_) on the surface of the brain that can be shorted together (green dashed line) to simulate an electrode with twice the surface area.

To provide a theoretical basis for this approach, we used a widely accepted electrical circuit model of the metal electrode (figure 1(A)) [30, 31]. In figure 1(B), two cortical surface electrodes (with impedances *Z*_e1_, *Z*_e2_) are each connected to an amplifier (with impedances *Z*_a1_, *Z*_a2_). The electrodes sense independent voltage sources in the neural tissue (*V*_s1_, *V*_s2_), which interact through the impedance of the brain tissue (*Z*_b1_, *Z*_b2_) and a shunt impedance between the two brain regions (*Z*_12_).

In the case with no shorting, each electrode measures the voltage in the underlying tissue, ${V_{{\text{e}}1}} \approx {V_{{\text{b}}1}}$ and ${V_{{\text{e}}2}} \approx {V_{{\text{b}}2}}$, assuming the input impedance of the amplifier is large. When the two electrodes are shorted, they measure a common voltage ${V_{\text{e}}}$ given by the equation:

\begin{equation*}{V_{\text{e}}} = \frac{{{Z_{{\text{e}}2}}}}{{{Z_{{\text{e}}1}} + {Z_{{\text{e}}2}}}}{V_{{\text{b}}1}} + \frac{{{Z_{{\text{e}}1}}}}{{{Z_{{\text{e}}1}} + {Z_{{\text{e}}2}}}}{V_{{\text{b}}2}}.\end{equation*}

The full derivation of this equation can be found in the appendix. If we assume the electrode impedances ${Z_{{\text{e}}1}}$ and ${Z_{{\text{e}}2}}$ to be equal, we find that the voltage measured at the surface is

\begin{equation*}{V_{\text{e}}} = \frac{{{V_{{\text{b}}1}} + {V_{{\text{b}}2}}}}{2}.\end{equation*}

This result suggests that, when two electrodes are shorted together, the voltage measured by the amplifiers is a linear combination of the voltages sensed by each individual electrode (*V*_b1_ and *V*_b2_). Because signals measured by iEEG electrodes are thought to reflect the average neural activity underneath them, this averaged activity should be equivalent to that sensed by a larger electrode covering the same cortical area as the two smaller electrodes. Here, we perform an experiment to directly test that hypothesis. If the hypothesis is true, physically shorting two adjacent electrodes can increase the effective surface area, thus providing a means to investigate the same region of neural tissue with electrodes of different sizes.

## 3. Methods

### 3.1. *In vitro* validation experiment

**Figure 2.**
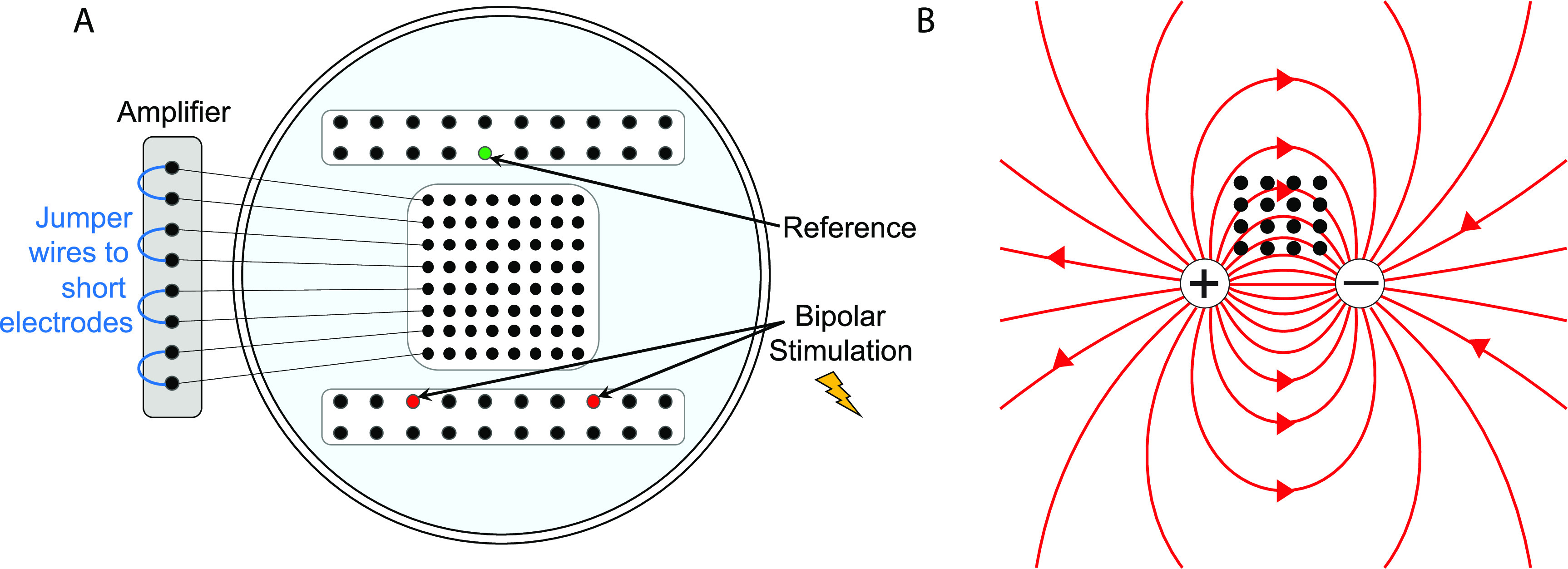
(A) Schematic of the agar gel disc with the 8 × 8 grid of electrodes, the 2 × 10 strip containing the reference electrode, and the 2 × 10 strip used for stimulation. An example is shown in which four pairs of electrodes are shorted at the amplifier using jumper wires. (B) The grid of intracranial electrodes (black dots) and the electric field lines of the dipole being sensed by the grid (red lines).

We performed an *in vitro* experiment to test the prediction from the circuit model that the signal recorded when electrodes are shorted together is equal to the average of the individual electrode signals (section 2). A disc of agar gel was used as the substrate because it has been shown to mimic both the structural and electrical properties of brain tissue [32]. The gel was mixed with water and NaCl to achieve a conductivity of ∼0.5 S m^−1^, to approximately match that of brain tissue [33]. For this experiment, we used three different types of electrode grids [1]: ‘Small’ electrodes (8 × 8 grid, 1.17 mm exposed diameter, 3 mm inter-electrode spacing, Ad-Tech FG64C-MP03X-000) [2], ‘Medium’ electrodes (4 × 4grid, 1.66 mm exposed diameter, 6 mm inter-electrode spacing, FG16C-SP06X-ORD—custom made), and [3] ‘Large’ electrodes (4 × 4 grid, 2.34 mm exposed diameter, 6 mm inter-electrode spacing, FG16C-SP06X-000). Note that the areas of the medium and large electrodes correspond to double and quadruple the area of the small electrodes, respectively. Each grid of intracranial electrodes was placed in the center of an agar gel disc (figure 2(A)). Bipolar electrical stimulation was applied to the gel substrate using a 2-by-10 electrode strip, and a second 2-by-10 strip was placed on the opposite side for use as an electrical reference. The stimulus was a sine wave of 350 *µ*V amplitude, with frequencies ranging from 10 to 70 Hz. The sinusoidally oscillating dipole created an electric field in the gel substrate that was sensed by the experimental grid of electrodes (figure 2(B)). Thirty seconds of data were recorded from these three grid types using Biopac EEG 100 C amplifiers and were sampled at 500 Hz, keeping the location of each grid on the substrate fixed relative to the stimulation electrodes.

**Figure 3.**
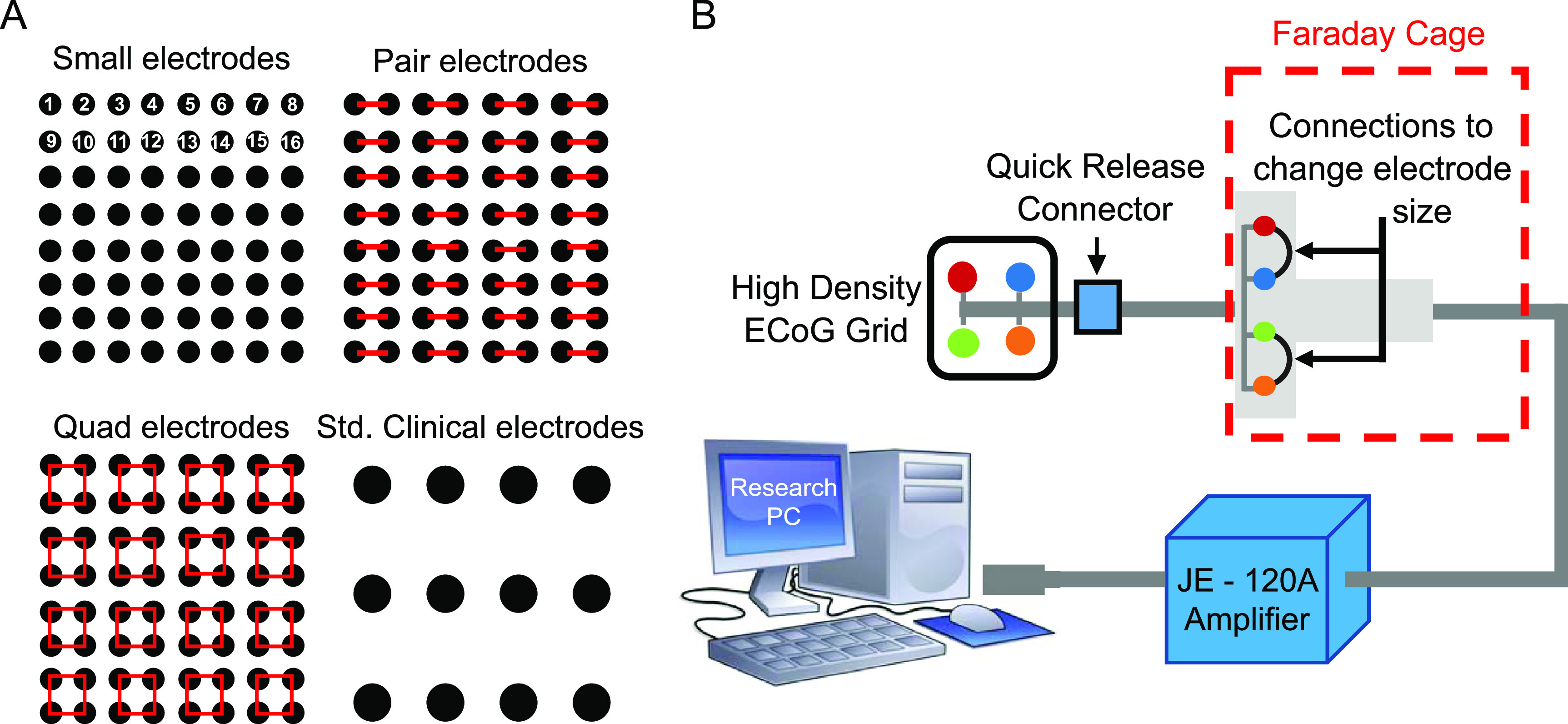
(A) Schematic that shows the shorting technique to create pair and quad surface areas, as well as a scale drawing of a standard grid size, for comparison. (B) Experimental setup, showing an example in which a small 2 × 2 grid (red, blue, green, and orange electrodes) are shorted together in pairs (red-blue and green-orange) using jumper wires.

After collecting data from every individual electrode on each grid during the first set of recordings, we reconfigured the recording setup for the small electrode grid by using jumper wires to electrically short specific sets of electrodes together (figure 2(A)). First, adjacent pairs of electrodes were shorted together to form the ‘pair’ configuration (*n* = 32 pair electrodes), and then 2-by-2 groups of four electrodes were shorted together to form the ‘quad’ configuration (*n* = 16 quad electrodes). The *in vivo* experiment in section 3.2 uses the same configurations (figure 3(A)). The bipolar stimulation described earlier was applied individually to each configuration, and the resulting electric field was measured at each pair and quad electrode.

Then, to verify that shorting adjacent electrodes mimics an electrode with larger surface area, we compared the signals obtained from the medium electrodes to the signals from the ‘pair’ recordings, and we compared the large electrode recordings to the ‘quad’ recordings. For each comparison, the electrodes being compared to one another had equivalent effective surface areas; if our hypothesis is correct, their recorded signals should also be equivalent. Further, we compared each of these sets of signals (medium vs. pair, large vs. quad) to the waveforms produced by mathematical averaging of the corresponding small electrode recordings. For example, we averaged pairs of signals from the small electrode recording and compared those to the physically shorted ‘pair’ electrode signals. We did an analogous comparison for the ‘quad’ signals. If the model in section 2 is valid, mathematical averaging, physical shorting, and the equivalent larger electrodes should all produce the same signal. We used a rank-based non-parametric approach (Wilcoxon rank sum test) to test the differences in root-mean-square (RMS) amplitude for the signals obtained from the various electrode configurations.

### 3.2. *In vivo* experiment

#### 3.2.1. Human data collection

The *in vivo* portion of this study was approved by the Institutional Review Board of the Children’s Hospital of Orange County (CHOC). Informed consent was obtained prior to involvement in the study. Three human subjects with medically intractable epilepsy were each implanted with a high-density 8 × 8 subdural grid of intracerebral EEG electrodes (Ad-Tech FG64C-MP03X-000) in the clinically determined seizure onset zone (SOZ) as part of phase 2 pre-surgical invasive monitoring. Patient information is given in table 1. Each electrode had an exposed surface area of 1.08 mm^2^ and electrode spacing was 3 mm center-to-center (this is the same grid used for the *in vitro* experiment in section 3.1, so we will similarly refer to these as the ‘small’ electrodes). The effective surface area was changed by electrically shorting adjacent electrodes in groups of two and four, thereby mimicking larger surface areas of 2.16 mm^2^ (‘pair’ electrodes) and 4.32 mm^2^ (‘quad’ electrodes), respectively (figure 3(A)). This was done by connecting jumper wires to the paired electrodes at the jack box outside the patient’s body (figure 3(B)). The jack box combines the individual electrode wires into an integrated cable before connecting to the amplifier. A quick-release connector enabled rapid reconfiguration of the electrode shorting, minimizing disruption to the patient’s recording. The jack box and jumper wires were placed in a Faraday cage to minimize electrical interference.

**Table 1.** Patient information. Age is indicated in years, and all subjects had focal epilepsy. The column for time post-implant indicates when the iEEG recordings were done, with Day 1 defined as the day of implantation.

Patient	Age at phase 2	Sex	Etiology	Outcome	Implanted electrodes	Time post-implant
1	15.4	F	Left frontal focal cortical dysplasia	Seizure-free	Subdural grids: two 2 × 4, one 4 × 8 and one HD 8 × 8	Day 2, evening
2	11.9	M	Unknown; two seizure foci (right frontal and right temporal)	Seizure-free	Subdural grids: one 8 × 8, one HD 8 × 8, one 2 × 4, two 1 × 6, one 4 × 5, one 1 × 4 Depth electrode: one 1 × 8	Day 2, evening
3	19	M	Left frontal traumatic brain injury	Seizure-free	Subdural grids: one HD 8 × 8, one 4 × 8, two 2 × 4, and one 2 × 8	Day 2, evening

Abbreviations: male (M), female (F), high-density (HD)

We collected 20 min iEEG recordings for each of three different electrode surface areas (small, pair, quad) from a grid in a static brain location while the subjects were sleeping. The sampling rate was 5 kHz, and the data were referenced to the common average of the 8 × 8 grid. The iEEG data were high pass filtered using a zero phase FIR filter at 1 Hz and notch filtered at 60 Hz, 120 Hz, and 180 Hz to remove electrical line noise before analysis. All analysis was done using custom code in MATLAB 2018b.

Similar to the *in vitro* study, we compared the results to the theoretical circuit model by generating ‘simulated pair’ electrode signals (consisting of the mathematical average of adjacent pairs of small electrodes) and ‘simulated quad’ electrodes (the mathematical average of four adjacent small electrodes). The averaging of signals was done on the raw iEEG data and the data were then re-referenced and filtered as described above. We compared the pair and quad electrode recordings to their associated simulated signals.

#### 3.2.2. Data analysis

##### 3.2.2.1. Correlation

To analyze how correlation between EEG signals changes with electrode size, we made two comparisons of correlation values: First, we compared small and pair electrodes with a vertical inter-electrode distance of 3 mm (e.g. correlation between electrodes 1 and 9 compared to the correlation between paired electrodes 1-2 and 9-10). Second, we compared pair and quad electrodes with a horizontal inter-electrode distance of 6 mm (e.g. correlation between electrodes 1-2 and 3-4 compared to the correlation between quad electrodes 1-2-9-10 and 3-4-11-12). The restriction on inter-electrode distance ensured that the comparison of correlation values was done between electrode pairs that had equivalent spacing. For each subject, five one-minute segments of data spaced at least two minutes apart were used for this analysis. The data were band pass filtered into three frequency bands using a zero phase FIR filter: low frequency (1–30 Hz), gamma-1 (30–60 Hz), and gamma-2 (60–100 Hz). In each frequency band, correlation values were calculated in five-second windows for pairs of channels and the correlation values were averaged over all time windows and electrode pairs for a given frequency band and electrode size. For each subject, the samples of size-specific mean correlation values were compared across electrode sizes using a Wilcoxon rank sum test. To assess the baseline distribution of correlation values (under the null hypothesis of zero correlation), correlation between channels was calculated using surrogate data. The surrogate data ${x_s}\left( t \right){\text{ }}$ was obtained by applying a random circular time shift to the original data $x\left( t \right)$ as follows:

\begin{equation*}{x_s}\left( t \right) = \left\{ \begin{array}{*{20}{r}} {x\left( {t + {t_r}} \right),{\text{ }}t &lt; N - {t_r}} \\ {x\left( {t - N + {t_r}} \right),{\text{ }}t \geqslant N - {t_r}} \end{array}\right.\end{equation*}

where ${t_{\text{r}}}$ is a random time point ranging from 1 to N−1.

##### 3.2.2.2. Amplitude

For each subject, the iEEG data from each of the three electrode configurations was bandpass filtered in the frequency range of 1–100 Hz. The bandpass filtered EEG was then divided into 100 segments of five seconds each. For each 5 s segment, the RMS value of the amplitude was calculated and then averaged across all channels. These samples of RMS amplitude values were compared across electrode sizes using a Wilcoxon rank sum test.

##### 3.2.2.3. Power spectrum

For every subject, power spectral density from 1 to 100 Hz was estimated for each of the 100 epochs of five seconds, for each electrode configuration. The following statistical analysis was done independently for each subject. To enable paired comparisons of the power spectra, the signals were grouped based on the quad electrode configuration. For example, small electrodes 1, 2, 9, and 10 were compared to two paired electrodes (1 shorted to 2, and 9 shorted to 10) and one quad electrode (1, 2, 9, and 10 shorted together) (see figure 3). For each set of four small electrodes, the power spectra were estimated by using the Fourier periodogram which is the data-analogue of the spectrum defined on the fundamental frequencies. Since periodograms are quite noisy, they need to be smoothed in order to obtain a mean-squared consistent estimator [34]. In some applications, it is more convenient to use log periodograms (rather than periodograms) because their variance is approximately constant across frequencies. Here, log periodograms were calculated and smoothed across frequencies using a moving average filter with a span of 5 data points (0.5 Hz). For each set of two pair electrodes, the two log periodograms were averaged and smoothed using a span of 10 data points (1 Hz). For the quad electrodes, the log periodograms were used without averaging and were smoothed using a span of 20 data points (2 Hz).

Thus, for each configuration, a set of 1600 log periodograms was obtained (16 signals × 100 epochs). To explore structures, patterns, and features in the sample of periodograms’ curves, we followed Ngo *et al* and constructed a functional box plot (FBP) [35], a generalization of the classical pointwise boxplot. For each curve, a modified band depth (MBD) value is computed [36]. This indicates whether or not a curve is covered by many pairs of curves in the data. Based on the ranks of MBD values, the FBP provides descriptive statistics, such as the functional median curve, which has the highest MBD value.

##### 3.2.2.4. Depth-based permutation test for the power spectrum

Let *F, G* and *L* be the distribution of log periodogram populations from three different settings (small, pair, and quad electrodes) with ${n_1} = {n_2} = {n_3} = 1600$. We propose a depth-based permutation test for our null hypothesis that the three populations of curves come from the same distribution, i.e. there is no difference in the distributions of the small, pair, and quad electrodes. Let $\left\{ {{x_1}, \ldots ,{\text{ }}{x_{{n_1}}}} \right\}$, $\left\{ {{y_1}, \ldots ,{\text{ }}{y_{{n_2}}}} \right\}$, and $\left\{ {{z_1}, \ldots ,{\text{ }}{z_{{n_3}}}} \right\}$ denote the three samples’ curves from distributions *F, G* and *L,* respectively.

Suppose that $R({x_1}), \ldots ,{\text{ }}R({x_{{n_1}}})$ are the corresponding ranks of $\left\{ {{x_1}, \ldots ,{\text{ }}{x_{{n_1}}}} \right\}$, measured by comparison to the combined three samples of size ${n_1} + {n_2} + {n_3}$. The test statistic is defined as $T = \sum\nolimits_{i = 1}^{{n_1}} {\text{rank}}\left[ {R\left( {{x_i}} \right)} \right]$, which is the sum of the MBD ranks in distribution $F$. Under the null hypothesis, *T* is the sum of ${n_1}$ numbers that are evenly distributed between 1 and ${n_1} + {n_2} + {n_3}$. If the alternative hypothesis is true, the sample ${x_i}$ will be more outlying than the other samples, which implies that the depth values will be smaller, with correspondingly smaller ranks. Thus, a small value of $T$ provides strong evidence to reject the null hypothesis. Since it is a challenging task to obtain the distribution of $T$ under the null hypothesis in a case of three samples, we then carry out a permutation test to compute the *p*-values, which is as follows:

(a) Permute electrode configuration labels (small, pair, and quad) among the samples in the combined set $\left\{ {{x_1}, \ldots ,{\text{ }}{x_{{n_1}}}} \right\}$ U $\left\{ {{y_1}, \ldots ,{\text{ }}{y_{{n_2}}}} \right\}$ U $\left\{ {{z_1}, \ldots ,{\text{ }}{z_{{n_3}}}} \right\}$ and denote the resulting samples of the $j$th permutation to be $\left\{ {x{^{^{\prime}}_{j1}}, \ldots ,{\text{ }}x{^{^{\prime}}_{j{n_1}}}} \right\}$, $\left\{ {y{^{^{\prime}}_{j1}}, \ldots ,{\text{ }}y{^{^{\prime}}_{j{n_2}}}} \right\}$, and $\left\{ {z{^{^{\prime}}_{j1}}, \ldots ,{\text{ }}z{^{^{\prime}}_{j{n_3}}}} \right\}$ for $j = 1, \ldots ,{\text{ }}J.$
(b) For each permutation, we compute the test statistic $T_j^{^{\prime}} = \sum\nolimits_{i = 1}^{{n_1}} {\text{rank}}\left[ {R\left( {x{^{^{\prime}}_{ji}}} \right)} \right].$
(c) The *p*-value is approximated by $\sum\nolimits_{j = 1}^J I$ $\left[ {T_j^{^{\prime}} &gt; {T_{{\text{obs}}}}} \right]\!/$ $J$ where ${T_{{\text{obs}}}}$ is the observed value of $T$ based on the original combined samples $\left\{ {{x_1}, \ldots ,{\text{ }}{x_{{n_1}}}} \right\}$ U $\left\{ {{y_1}, \ldots ,{\text{ }}{y_{{n_2}}}} \right\}$ U $\left\{ {{z_1}, \ldots ,{\text{ }}{z_{{n_3}}}} \right\}$, and $I{\text{ }}$ is the indicator function.

Because the different electrode configurations were recorded at different times, we also tested whether the power spectra were stable over time. For each configuration, we compared the power spectra in the first five minutes of the recording to those in the last five minutes, using five equally spaced 25 s intervals for each case. Depth based permutation testing was done as described above on the two sets of five curves in each scheme.

#### 3.2.3. Analysis of interictal spikes

We also wanted to characterize the impact of electrode size on the morphology of transient electrographic events. Because the study subjects had refractory epilepsy, we focused on interictal epileptiform discharges, i.e. interictal spikes. For this analysis, 20 min segments of data were used, each one clipped from the long-term recording while the patient was sleeping, between midnight and 12:30 am. Interictal spikes were manually marked in the iEEG data in the small electrode configuration for each subject under the supervision of a board-certified epilepsy specialist (Daniel Shrey). We then simulated each spike in the pair and quad electrode configurations by mathematically averaging the corresponding small electrode data. We defined the SNR of each spike as the signal to background amplitude ratio. The amplitude of the spike was measured as the difference between the minimum and maximum voltages recorded over the duration of the spike. To calculate the background amplitude, a one-second interval around the spike, not containing the spike, was considered. The signal in this window was rectified, and the average of the rectified signal was defined as the baseline amplitude. The SNRs were compared across the three electrode configurations and each spike was classified into one of three types: type S, in which a small electrode had the highest SNR, type P, in which a pair electrode had the highest SNR, and type Q, in which a quad electrode had the highest SNR. The spatial spread, defined as the combined area of the electrodes in which the SNR of the signal exceeded 1.5 during the time of the spike, was also calculated for each spike.

## 4. Results

### 4.1. *In vitro* study: physical shorting of electrodes mimics larger electrode sizes

**Figure 4.**
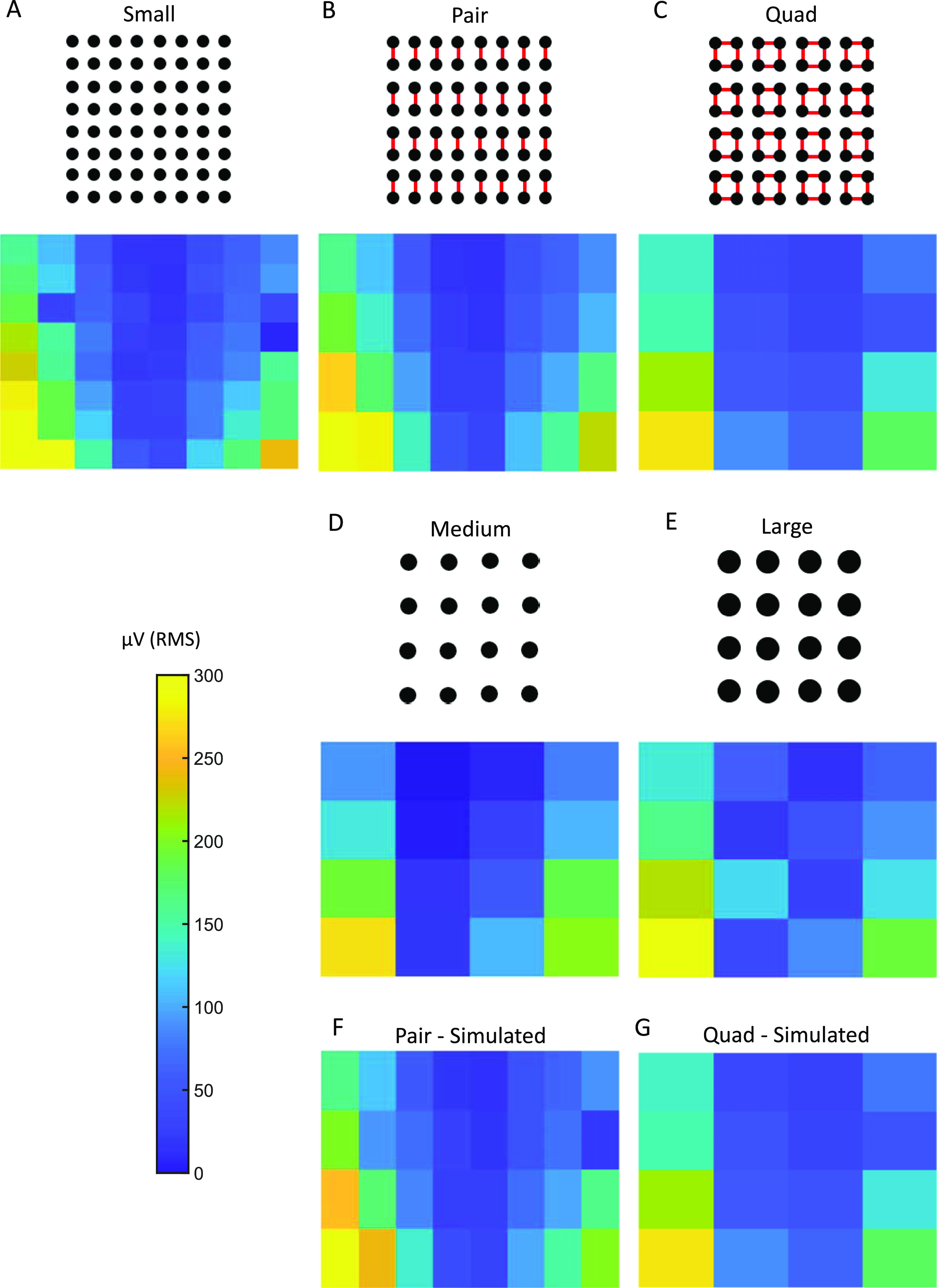
RMS amplitude values measured using (A) small electrodes, (B) pair electrodes via physical shorting, (C) quad electrodes via physical shorting, (D) medium electrodes, in which each electrode has a surface area equivalent to a pair electrode, and (E) large electrodes, in which each electrode has a surface area equivalent to a quad electrode. RMS amplitude values measured using (F) simulated pair and (G) simulated quad electrodes, which were obtained by mathematically averaging data from small electrodes. For each electrode, the RMS amplitude was calculated using the entire 30 s recording.

The *in vitro* experimental results are shown in figure 4. For the small, medium, and large, as well as the pair and quad electrodes, the measured signal maintained the original sinusoidal shape and frequency of stimulation for all stimuli. The RMS amplitudes varied according to the electric field created by the bipolar stimulation (figure 4). For all electrode configurations, the highest amplitude was observed at the boundary of the grid, and the amplitude decreased for the inner electrodes. Amplitude also decreased as the vertical distance from the stimulating electrodes increased. These results are consistent with the electric field lines in figure 2(B). We found that the amplitudes were highest for the smallest electrodes and decreased with an increase in electrode size, and this effect was also seen in the shorted electrodes.

The RMS amplitudes of the signals from the pair and medium electrodes were not statistically different from one other (Wilcoxon rank sum test, *p*-value >0.5, figures 4(B) and (D)). This was also true in the case of quad and large electrodes (figures 4(C) and (E)). These results indicate that shorting electrodes together mimics larger surface areas, consistent with our hypothesis. The lack of an exact correspondence between the amplitude values for different electrode configurations can be attributed to the limitations of the experimental setting, including slight variations in the positioning of the electrode grids and the fact that the adjacent shorted electrodes are not contiguous. For each electrode configuration, we changed the stimulation frequency from 10 to 70 Hz and found no difference in RMS values.

For both pair and quad recordings, the RMS values from the mathematically averaged signals were not significantly different from the corresponding RMS values obtained by physically shorting the electrodes (figure 4(F) compared to figure 4(B), and figure 4(G) compared to figure 4(C); Wilcoxon rank sum test, *p*-value >0.5).

### 4.2. *In vivo* study

Data were collected from three human subjects (1 female, 2 male) aged 15.4, 11.9, and 19 years. Board-certified epilepsy specialists verified that the use of the high-density subdural grid did not impede their ability to clinically interpret the iEEG signals or identify the electrodes where seizure activity first began. In one subject, functional mapping was also successfully performed using the high-density subdural grid [37].

#### 4.2.1. EEG correlation does not depend on electrode size

**Figure 5.**
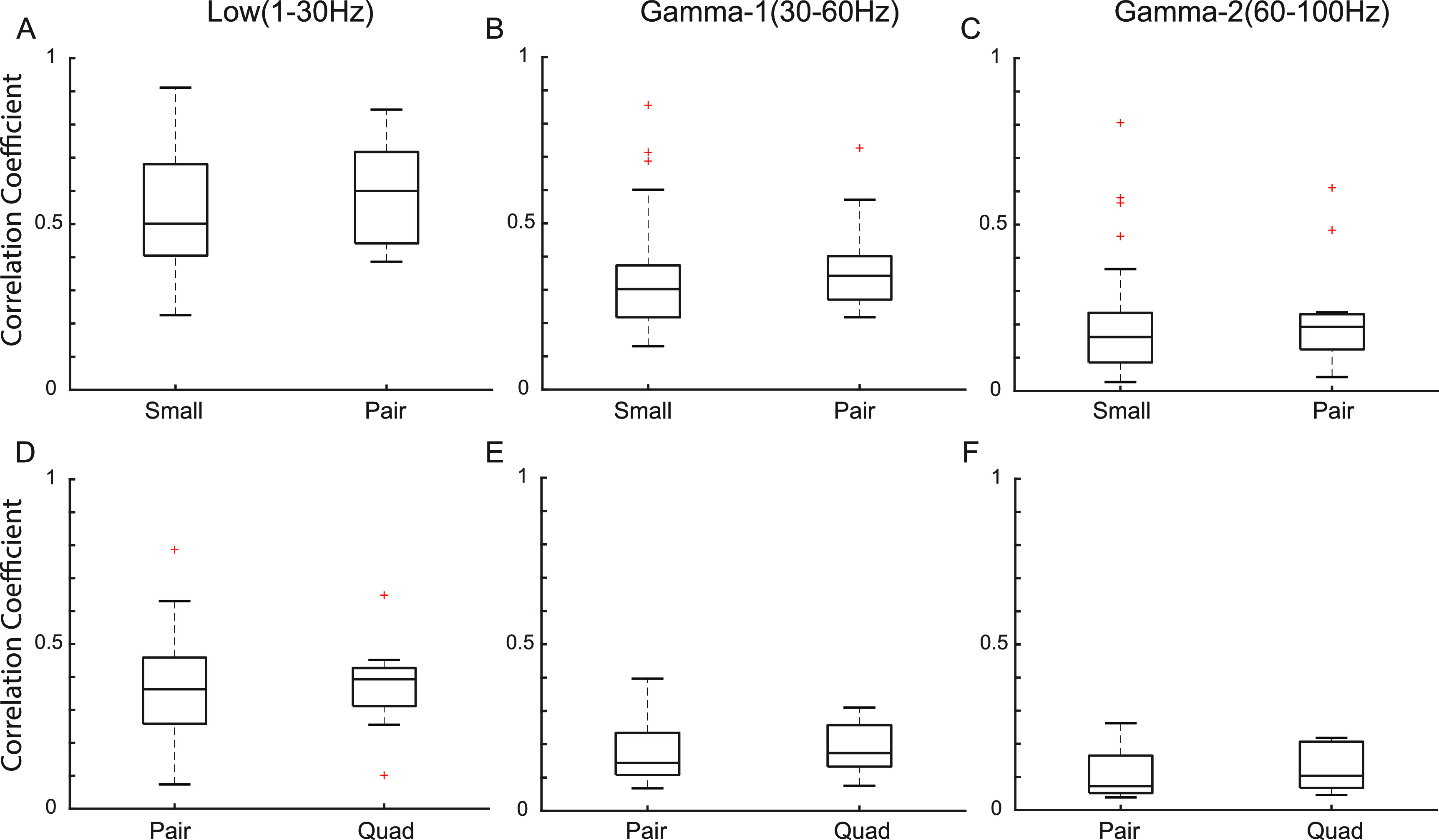
(A)–(C) Boxplots of EEG correlation values for electrodes at a fixed distance for small and pair electrode sizes (*n* = 32 for small and *n* = 16 for pair). (D)–(F) Boxplots of EEG correlation values for electrodes at a fixed distance for pair and quad electrode sizes (*n* = 16 for pair and *n* = 8 for quad). The box plots are generated from data points corresponding to the average correlation across all five-second time windows for the electrode pairs from one subject. Results are shown for the low (left), Gamma-1 (middle), and Gamma-2 frequency bands (right). Data are shown for a single representative subject; data for the other two subjects can be found in supplementary figures S1 and S2.

**Figure S1.**
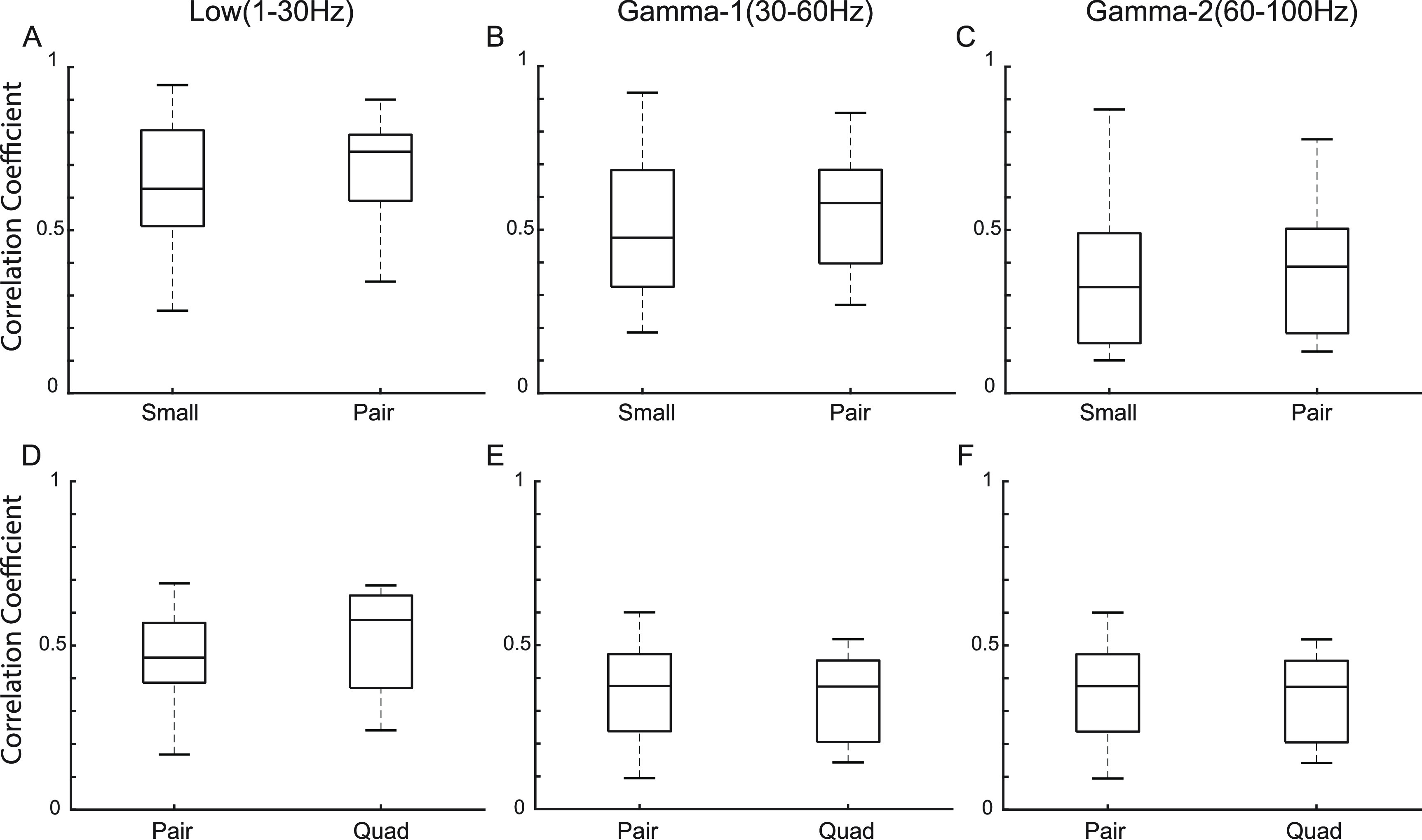
(A)–(C) Boxplots of EEG correlation values for electrodes at a fixed distance for small and pair electrode sizes (*n* = 32 for small and *n* = 16 for pair) for subject 1. (D)–(F) Boxplots of EEG correlation values for electrodes at a fixed distance for pair and quad electrode sizes (*n* = 16 for pair and *n* = 8 for quad). The box plots are generated from data points corresponding to the average correlation across all five-second time windows for the electrode pairs from one subject. Results are shown for the low (left), Gamma-1 (middle), and Gamma-2 frequency bands (right).

**Figure S2.**
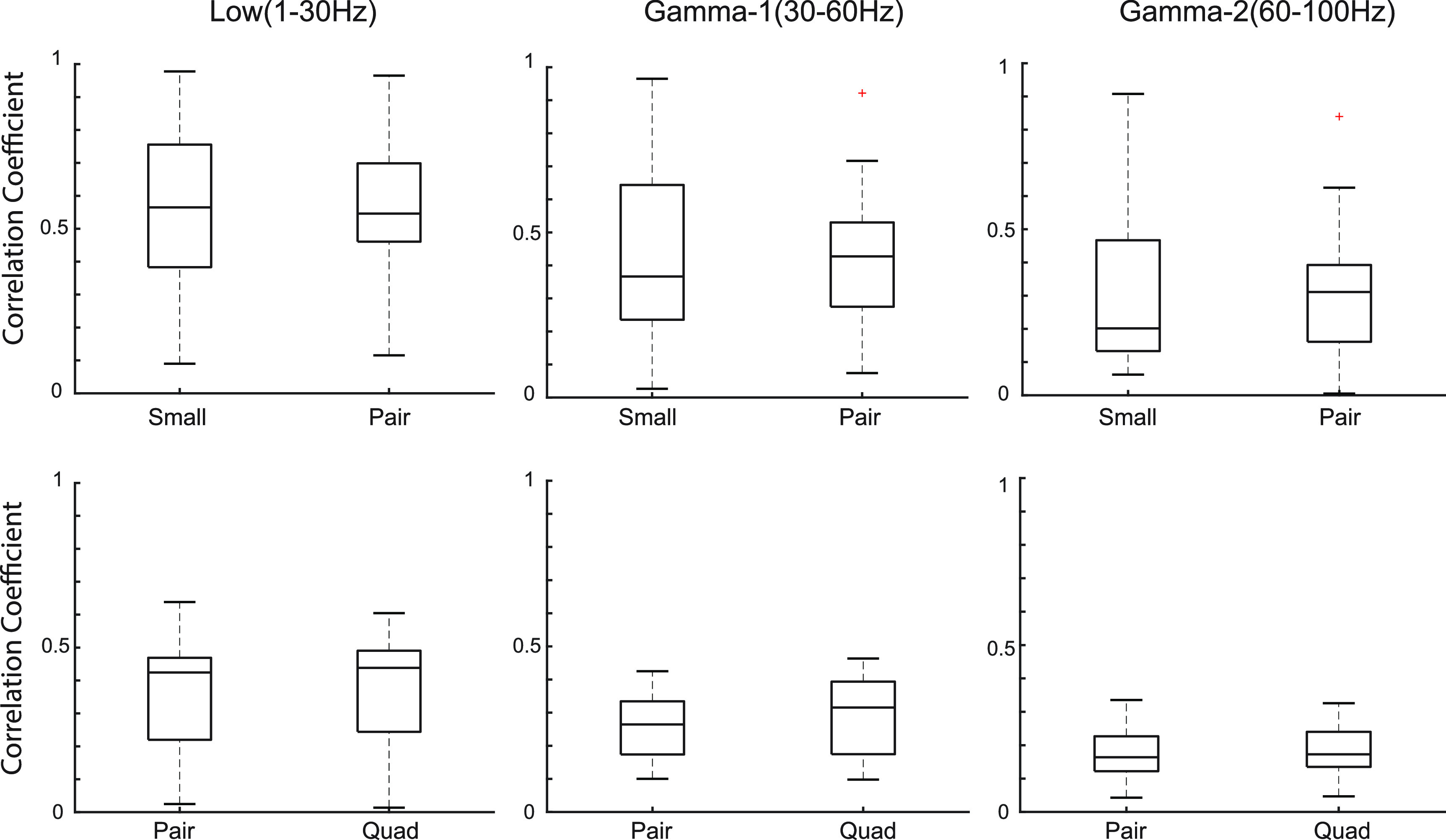
(A)–(C) Boxplots of EEG correlation values for electrodes at a fixed distance for small and pair electrode sizes (*n* = 32 for small and *n* = 16 for pair) for subject 3. (D)–(F) Boxplots of EEG correlation values for electrodes at a fixed distance for pair and quad electrode sizes (*n* = 16 for pair and *n* = 8 for quad). The box plots are generated from data points corresponding to the average correlation across all five-second time windows for the electrode pairs from one subject. Results are shown for the low (left), Gamma-1 (middle), and Gamma-2 frequency bands (right).

In the human iEEG recordings, we calculated the correlation between EEG signals from electrodes at a fixed distance. We found no statistically significant differences in correlation when comparing small and pair electrodes or pair and quad electrodes, in any frequency band or subject (figure 5 and supplementary figures S1 and S2). However, we did note a trend of increasing correlation with increasing electrode size. The correlation values in all three frequency bands in all three subjects were significantly higher than the baseline correlation values calculated using the time shifted surrogate data (range: −0.02 to 0.01).

#### 4.2.2. EEG amplitude and power decrease with increasing electrode size

**Figure 6.**
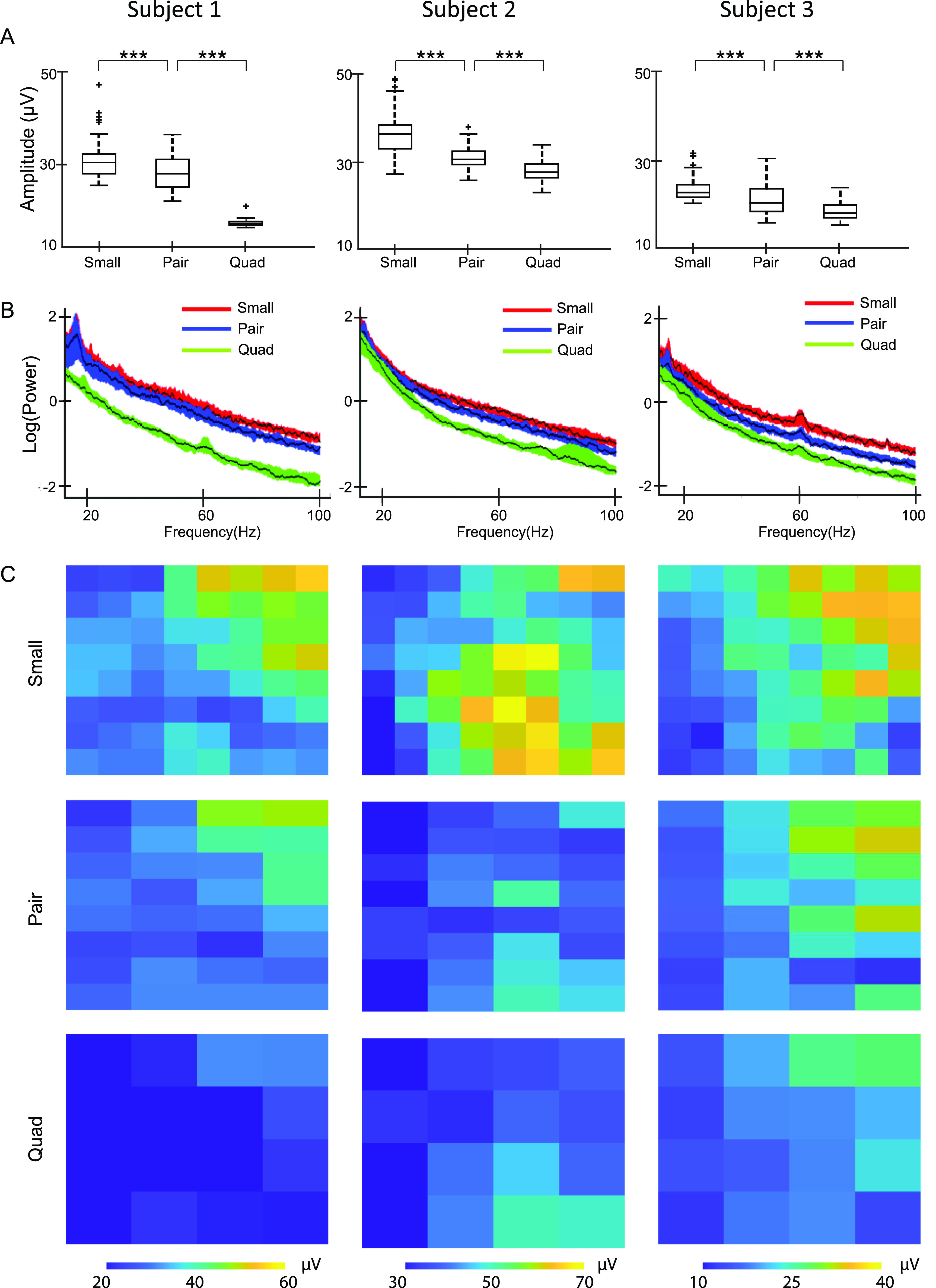
(A) Box plots of iEEG RMS amplitude in the 1–100 Hz frequency band measured using electrodes of three different sizes in the three subjects. Data from each subject are shown in a single column, across all subfigures. (B) Functional median curves for the power spectra in all three subjects. The black lines show the median curves, and the colored areas denote the 50% central region. (C) Heat maps of average RMS amplitude values for all electrodes in each subject measured using small, pair, and quad electrodes (physically shorted) in the 1–100 Hz band. For example, the results for the small electrodes show values in an 8 × 8 grid, with each colored square representing one electrode. For each electrode, the RMS amplitude was calculated for 5 s intervals and then those values were averaged. Each column of subfigures corresponds to the results for one subject and the subfigures in each row correspond to small, pair, and quad electrodes. *indicates *p*-values <0.05, ** indicates *p* < 0.01, *** indicates *p* < 0.001. All *p*-values are corrected for multiple comparisons using the Bonferroni method.

The iEEG RMS amplitude was highest for the small electrodes, and it decreased as electrode size increased (figure 6(A)). The pair and quad electrode sizes were created via physical shorting of small electrodes. The differences were statistically significant for all three electrode sizes across all three subjects.

The power spectra for the three electrode sizes overlapped in the low frequencies, based on the 50% central regions of the curves, but they were distinguishable for higher frequencies (figure 6(B)). Overall, the median periodogram curves were higher for small electrodes, which indicates that the iEEG power is higher for small electrodes compared to larger ones. Among the 48 sets of channels analyzed (16 from each subject), 39 showed a significant difference in the power spectra between the three different electrode sizes using the depth-based permutation test (*p* < 0.05).

Note that there is significant spatial variation of the iEEG amplitude across the subdural grid (figure 6(C)), possibly related to differences between tissue inside and outside the seizure onset zone. In all three subjects, the clinical team localized the seizure onset to an electrode on the high-density subdural grid. However, for each patient, the spatial distribution of amplitudes was consistent across electrode sizes, with amplitude generally decreasing as electrode size increased. As in the *in vitro* experiment, the amplitude in the larger electrodes appeared to be consistent with the average of the corresponding small electrodes (figure 6(C)).

#### 4.2.3. Interictal spike morphology depends on the size and location of the neural generator, relative to electrode size

To quantify the impact of electrode size on interictal spike morphology, we measured the spike SNR as a function of electrode size. We were unable to do direct event-wise comparisons using the physically shorted electrodes because the data for each electrode size were recorded at different times. Therefore, we used simulated spikes to estimate the change in SNR as electrode size varied. We first marked a total of 500 spikes, using the data from the small electrodes from all three subjects. For each spike, we then calculated simulated spikes in pair and quad electrodes, via mathematical averaging of the associated small electrode iEEG.

**Figure 7.**
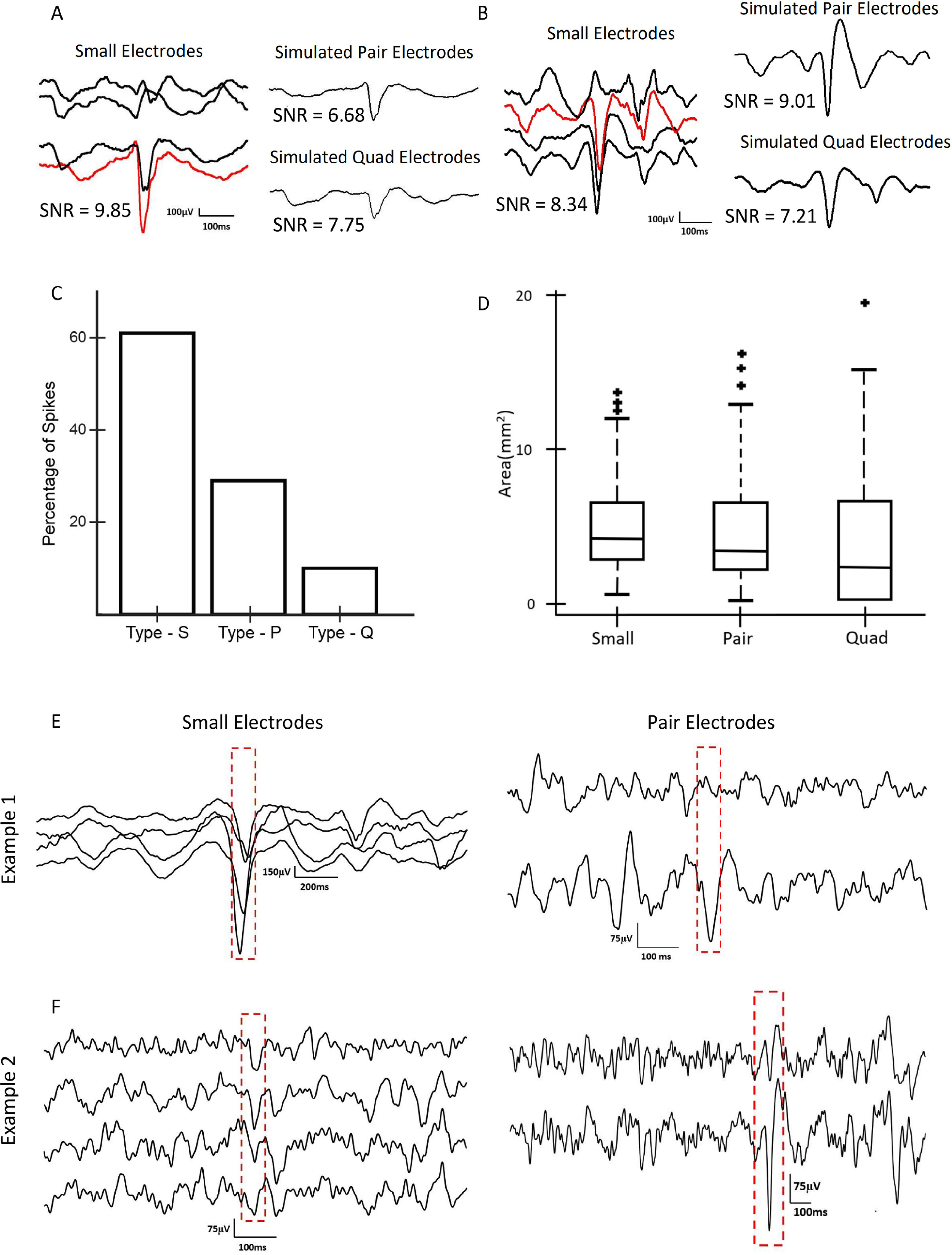
(A) Simulation results showing an example of a type S interictal spike, in which the SNR is high for a small electrode and lower for a pair electrode simulated via mathematical averaging. (B) Simulation results showing an example of a type P spike, in which the SNR for simulated pair electrodes is higher than for small electrodes. (C) Bar graph showing the percentages of type S, type P, and type Q spikes for all three subjects. Data for pair and quad electrodes were obtained via mathematical averaging of small electrodes. (D) Spatial spread of spikes for different electrode sizes across all three subjects. Data for pair and quad electrodes were obtained via mathematical averaging of small electrodes. (E) Example of interictal spikes captured via physical shorting of electrodes, in which a high SNR spike is visible in the small electrodes (left) and a low SNR spike is seen when the same brain region is later recorded using pair electrodes (right). (F) A second example of an interictal spike captured via physical shorting of electrodes. Here, a spike with low SNR is visible in the small electrodes (left); when physical shorting was later used to record from pair electrodes, larger SNR spikes were noted in the same set of channels (right).

Two examples of simulated spikes demonstrate why the smallest electrodes are not always associated with the highest SNR. In the first case (figure 7(A)), the spike is clearly localized to a single electrode. Therefore, averaging reduces the SNR of the spike, and the biggest SNR is observed in the smallest electrode. In the second case (figure 7(B)), the spike is more widespread, and averaging increases the amplitude relative to the background. Consequently, the largest SNR is observed for the pair electrode.

Across all visually-marked spikes, 61% had the highest SNR in the small electrodes, 29% had the highest SNR in the simulated pair electrodes, and 10% had the highest SNR in the simulated quad electrodes (figure 7(C)). When measuring with small electrodes, the spikes were seen in a larger number of channels, but the cortical surface area of the spikes remained approximately constant across electrode sizes (figure 7(D), *p* > 0.1).

Anecdotally, when visually comparing the interictal spikes from the three electrode sizes, obtained via physical shorting and recorded at independent time points, we found examples that were consistent with these results. Figure 7(E) shows an example of prominent spikes recorded by small electrodes that exhibited lower amplitude when the same brain location was later recorded with physically-shorted pair electrodes, analogous to the simulation results in figure 7(A). A second example shows a case where the spikes had low amplitude when recorded with small electrodes, but they became more visually prominent when the same brain region was later recorded with physically-shorted pair electrodes (figure 7(F)), analogous to the simulated data in figure 7(B).

## 5. Discussion

Through this study, we have introduced a method for dynamic selection of the size of iEEG electrodes after implantation in the human brain. We first presented an electrical circuit model, then performed an *in vitro* validation of that model, showing that there were no significant differences between physically shorted electrodes and larger electrodes with equivalent surface areas. The signals from physically shorted electrodes were also consistent with simulated signals obtained by mathematically averaging the signals from the small electrodes. In human subjects, we found that increasing electrode size leads to lower iEEG power and amplitude, but no difference in correlation between channel pairs. The morphology of interictal spikes was also impacted by electrode size and depended on the size and location of the neural generator relative to the electrodes.

The development of this novel recording technique is significant because it will enable direct tests of the impact of electrode size on transient electrographic events, such as epileptiform discharges and HFOs, as well as seizure localization and functional mapping. This may impact the design and manufacture of Food and Drug Authority (FDA) approved intracranial electrodes for human use, as well as clinical procedures for patient evaluation. Moreover, our data suggest that mathematical averaging of iEEG electrodes is consistent with physical shorting of the same electrodes; if this hypothesis is further validated, such studies could be done using a single high-density grid and simple mathematical averaging, which would greatly increase the flexibility and applicability of this technique.

Our results shed light on hypotheses put forward in previous literature. In an *in vivo* study of rat somatosensory cortex, the amplitudes of sensory evoked potentials recorded using small electrodes were higher than those recorded using larger electrodes [38]. Our results in figure 6 show the same trend. In our simulations of interictal spikes, we estimated that a majority of spikes would have the highest SNR when recorded with small electrodes, but approximately 40% of spikes had higher SNR values when simulated in pair and quad recordings, using mathematical averaging. Anderson *et al* posited that using larger electrodes for recording action potentials of neurons decreases the SNR. Assuming that the spatial extent of the action potential is small relative to the electrode size, this is consistent with our observations of interictal spikes (figure 7) [18]. While single neuron spikes occur on a much smaller spatial scale than interictal epileptiform discharges, the two cases share conceptual similarities. Lastly, in a study that measured correlation using both micro-ECoG and standard ECoG electrodes, the larger electrodes exhibited higher correlation [22]. Wang *et al* also reported higher degrees of dependence between larger electrodes [21]. Although our results show a trend of an increase in correlation with electrode size (figure 5), this difference was not found to be significant for the sizes compared. This could be because our analysis was done at a fixed inter-electrode distance, while the inter-electrode distance varied with electrode size in the prior studies.

There are some limitations to this study. The electrodes in the ECoG grids had a center-to-center distance of 3 mm, so the adjacent shorted electrodes were not contiguous. That is, there was some area of tissue between shorted electrodes that was not in contact with them, which is a deviation from the assumption of a single, continuous electrode. Using tripolar concentric ring electrodes [39] or decreasing the interelectrode distance in the grid would help alleviate this limitation. However, because this study was done on human subjects, we were limited to the use of FDA approved electrodes. Another limitation was that the recordings from electrodes of different sizes were done sequentially and, therefore, were obtained at different times. We repeated these measurements to verify that our findings remained robust and were stable over time. In particular, we found no significant difference in the value of the power spectrum when comparing data spaced 10 min apart, for any electrode size. Additionally, the data in this study were obtained from patients with epilepsy, and this disease is known to alter various features of the iEEG data. Because our aim was to study the effects of electrode size on iEEG, irrespective of the origin of the activity, we believe this did not significantly impact our results. Moreover, all comparisons were made using data recorded from the same region of brain tissue; therefore, the presence or absence of epileptogenic activity should impact all conditions equally.

Future work in this field may benefit from the use of modeling techniques like finite element modeling (FEM). FEM based methods have been used to numerically solve the EEG forward problem accurately, that is, to determine the voltages at the surface of the brain given the location of deep sources. This is done by incorporating complex geometries and electrical properties of the brain into the model [40]. FEM could be applied to our study to exactly simulate electrodes of different sizes having similar geometries and spacing between them. This could address the inconsistency between our simple mathematical averaging approach and the non-contiguous area of the larger electrodes in this study. Thus, an FEM-based approach could provide a more accurate mathematical model of the measured electrical activity, as a function of electrode geometry and spacing, given a set of neural sources.

This study is the first to present a method to record intracranial EEG from a static section of neural tissue using electrodes of different effective sizes. This technique provides an avenue for multi-scale analysis of neurological phenomena recorded from a single location in the brain. The methods used here could also enable dynamic selection of optimal electrode sizes for detection of neurological events like seizures, HFOs, and interictal spikes, as well as recordings used by neural prostheses or BCIs. This is especially relevant in a clinical setting where the precise locations of these events are unknown prior to surgery and are highly variable across patients. Clinicians rely on visual analysis of the iEEG, and electrographic events that are barely visible in data from a particular electrode size could be more accurately studied when measured with a larger or smaller electrode. In applications like neural prostheses and BCI where the quality of the signals is paramount, our methods can be used to maximize SNR while requiring only a single implantation of a standard commercially available electrode grid. Overall, this technique has the potential to facilitate patient-specific optimization of iEEG recordings, for both clinical and engineering applications.

## Data Availability

The data that support the findings of this study are available upon reasonable request from the authors.

## Appendix

### Circuit model calculations

Please refer to figure 1(B) for a diagram of the electrical circuit.

The voltage observed at the brain surface (${V_{{\text{b}}1}}$ and ${V_{{\text{b}}2}}$) due to two sources ${V_{{\text{s}}1}}$ and ${V_{{\text{s}}2}}$ can be calculated from the circuit, considering only the part to the left of the red line. We get the voltages to be:

\begin{equation*}{V_{{\text{b}}1}} = \frac{{{Z_{{\text{b}}2}} + {Z_{12}}}}{{{Z_{{\text{b}}1}} + {Z_{{\text{b}}2}} + {Z_{12}}}}{V_{{\text{s}}1}} + \frac{{{Z_{{\text{b}}1}}}}{{{Z_{{\text{b}}1}} + {Z_{{\text{b}}2}} + {Z_{12}}}}{V_{{\text{s}}2}}\end{equation*}

\begin{equation*}{V_{{\text{b}}2}} = \frac{{{Z_{{\text{b}}2}}}}{{{Z_{{\text{b}}1}} + {Z_{{\text{b}}2}} + {Z_{12}}}}{V_{{\text{s}}1}} + \frac{{{Z_{12}} + {Z_{{\text{b}}1}}}}{{{Z_{{\text{b}}1}} + {Z_{{\text{b}}2}} + {Z_{12}}}}{V_{{\text{s}}2}}.\end{equation*}

When the recording electrodes are added to the circuit (right of the red line), the voltages sensed at each electrode (${V_{{\text{e}}1}}$ and ${V_{{\text{e}}2}}$) will be approximately equal to ${V_{{\text{b}}1}}$ and ${V_{{\text{b}}2}}$ respectively, considering the input impedance of the amplifier to be very large.

Then, for example, if we assume equal tissue impedances ${Z_{{\text{b}}1}},{Z_{{\text{b}}2}}$ and ${Z_{12}}$, the voltage sensed at each electrode would be,

\begin{equation*}{\text{ }}{V_{e1}} = \frac{2}{3}{V_{s1}} + \frac{1}{3}{V_{s2}}\end{equation*}

\begin{equation*}{V_{{\text{e}}2}} = \frac{1}{3}{V_{{\text{s}}1}} + \frac{2}{3}{V_{{\text{s}}2}}.\end{equation*}

To represent an electrode with twice the surface area, we model two adjacent electrodes that are shorted together (green dashed line). In this case, the voltages at the brain surface are

\begin{align*}{V_{{\text{b}}1}} &amp; = \frac{{{Z_{{\text{b}}2}}\left( {{Z_{\text{e}}} + {Z_{12}}} \right) + {Z_{12}}{Z_{\text{e}}}}}{{\left( {{Z_{\text{e}}} + {Z_{12}}} \right)\left( {{Z_{{\text{b}}1}} + {Z_{{\text{b}}2}}} \right) + {Z_{12}}{Z_{\text{e}}}}}{V_{{\text{s}}1}}\nonumber\\[3pt] &amp;\quad + \frac{{{Z_{{\text{b}}1}}\left( {{Z_{\text{e}}} + {Z_{12}}} \right)}}{{\left( {{Z_{\text{e}}} + {Z_{12}}} \right)\left( {{Z_{{\text{b}}1}} + {Z_{{\text{b}}2}}} \right) + {Z_{12}}{Z_{\text{e}}}}}{V_{{\text{s}}2}}\end{align*}

\begin{align*}{V_{b2}}&amp; = \frac{{{Z_{\text{b}2}}\left( {{Z_{\text{e}}} + {Z_{12}}} \right)}}{{\left( {{Z_{\text{e}}} + {Z_{12}}} \right)\left( {Z_{{\text{b1}}}} + {Z_{{\text{b2}}}} \right) + {Z_{12}}{Z_{\text{e}}}}}{V_{\text{s}1}} \nonumber\\[3pt] &amp;\quad + {\text{ }}\frac{{{Z_{\text{b1}}}\left( {{Z_{\text{e}}} + {Z_{12}}} \right) + {Z_{12}}{Z_{\text{e}}}}}{{\left( {{Z_{\text{e}}} + {Z_{12}}} \right)\left( {{Z_{\text{b}1}} + {Z_{\text{b2}}}} \right) + {Z_{12}}{Z_{\text{e}}}}}{V_{\text{s2}}}\end{align*}

where ${\text{ }}{Z_e}$=${Z_{{\text{e}}1}}$ + ${Z_{{\text{e}}2}}$

Note that the expressions for ${V_{{\text{b}}1}}$ and ${V_{{\text{b}}2}}$ become approximately equal to the un-shorted case when the electrode impedance is small compared to the tissue impedance.

Finally, the common voltage sensed by the two shorted electrodes (${V_{{\text{e}}1}}$ = ${V_{{\text{e}}2}}$ = ${V_{\text{e}}}$) is given by:

\begin{equation*}{V_{\text{e}}} = \frac{{{Z_{{\text{e}}2}}}}{{{Z_{{\text{e}}1}} + {Z_{{\text{e}}2}}}}{V_{{\text{b}}1}} + \frac{{{Z_{{\text{e}}1}}}}{{{Z_{{\text{e}}1}} + {Z_{{\text{e}}2}}}}{V_{{\text{b}}2}}.\end{equation*}

If we assume equal electrode impedances ${Z_{{\text{e}}1}}$ = ${Z_{{\text{e}}2}}$, then we obtain,

\begin{equation*}{V_e} = \frac{{{V_{b1}} + {\text{ }}{V_{b2}}{\text{ }}}}{2}\end{equation*}

This is the average of the two voltages from the individual electrodes before shorting. Therefore, this model suggests that physical shorting of adjacent electrodes is equivalent to mathematical averaging of the activity recorded by the individual electrodes.

### Impedances of electrodes in the *in-vitro* study

**Table:**

Configuration	Impedance (kohms, measured at 70 Hz)
Small	30–40
Medium	10–20
Pair	20–30
Large	5–10
Quad	10–20
